# The holy grail of longevity research

**DOI:** 10.7554/eLife.85001

**Published:** 2022-12-23

**Authors:** Ajay S Mathuru

**Affiliations:** 1 Yale-NUS College Singapore Singapore; 2 The Institute for Digital Medicine (WisDM), Yong Loo Lin School of Medicine, National University of Singapore Singapore Singapore; 3 Department of Physiology, Yong Loo Lin School of Medicine, National University of Singapore Singapore Singapore; 4 Institute of Molecular and Cell Biology, A*STAR Singapore Singapore

**Keywords:** Nothobranchius furzeri, african killifish, turquoise killifish, N. furzeri, Other

## Abstract

A new technology to study physiology and cognition elevates African turquoise killifish as a model organism for studies of aging in vertebrates.

**Related research article** McKay A, Costa EK, Chen J, Hu CK, Chen X, Bedbrook CN, Khondker RC, Thielvoldt M, Priya Singh P, Wyss-Coray T, Brunet A. 2022. An automated feeding system for the african killifish reveals effects of dietary restriction on lifespan and allows scalable assessment of associative learning. *eLife* **11**:e69008. doi: 10.7554/eLife.69008.

Aging is a major risk factor for numerous chronic diseases, such as dementia, metabolic syndromes, and cancers, (Hou et al., 2019; Kennedy et al., 2014; Niccoli and Partridge, 2012), and age-related declines in health are poised to become significant economic and clinical challenges (European Commission, 2014). As a consequence, researchers, governments and drug companies have been trying to identify how aging is influenced by lifestyle choices and by biological, environmental and socio-economic factors (Crane et al., 2022). A key challenge is to develop innovative approaches that can help us to better understand the biology of aging and to accurately quantify age-dependent changes in physiology and cognition. The latter is necessary to evaluate the costs and benefits of potential interventions. Now, in eLife, Anne Brunnet (Stanford University) and colleagues – including Andrew McKay, Emma Costa and Jingxun Chen as joint first authors – report a fresh dimension to this quest (McKay et al., 2022).

Finding appropriate animal models is crucial in biomedicine, but it is rare for a single species (such as mice) to have all the characteristics required and also capture all the aspects of a target species (such as humans). The observation that “all models are wrong, but some are useful” captures this concept succinctly (even if it was first made about statistical models, not animal models; Box, 1976). However, it is possible to overcome this limitation by having a diverse pool of animal models that can help uncover fundamentally conserved phenomena and fuel innovative thinking (Mathuru et al., 2020). This is particularly relevant for aging studies, where longevity can be affected by species-specific adaptations and dramatically divergent evolutionary trajectories.

McKay et al. showcase new technologies and resources for the African turquoise killifish, *Nothobranchius furzeri* (Figure 1A). Compared to other vertebrate model organisms, killifish have an extremely short life cycle, during which they go through all the stages of life – from a larva to a senile adult – within a few weeks (Harel et al., 2015; Reichard and Polačik, 2019; Terzibasi Tozzini and Cellerino, 2020; Valenzano et al., 2015).

**Figure 1.**
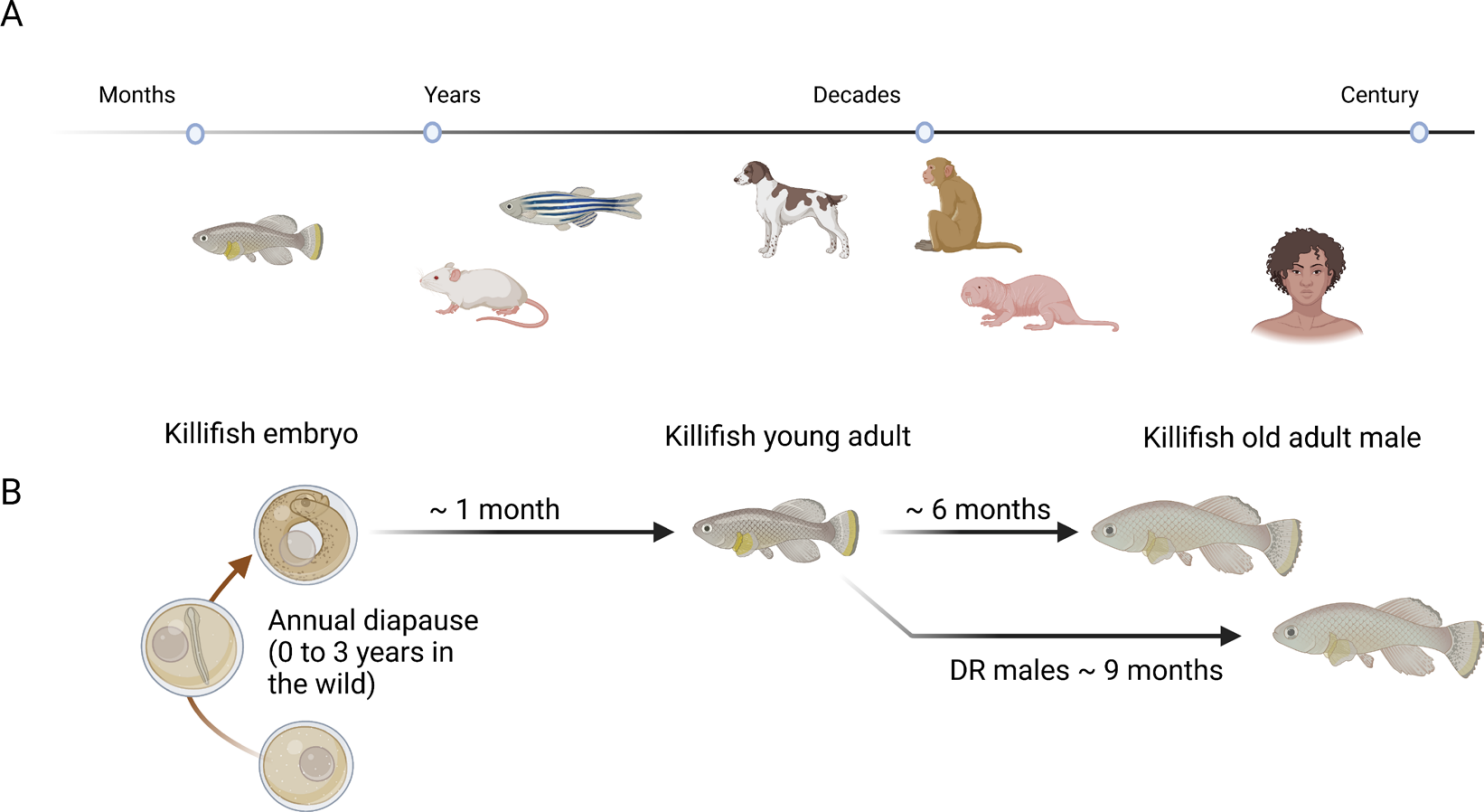
Killifish as a model organism to study aging. (**A**) Of the various model organisms used to study aging and longevity in vertebrates, killifish (left) have the shortest lifespans. Other model organisms used to study aging and longevity in vertebrates include (in order of increasing lifespan) mice, zebrafish, dogs, macaques, naked mole rats and humans. (**B**) In the wild, killifish produce desiccation-resistant eggs and can pause egg development if environmental conditions are unfavorable. Once development resumes, killifish complete their lifecycle within six months. McKay et al. demonstrate that a restricted diet can extend the lifespan of male killifish (DR males) by up to three months.

Image created with BioRender.com.

For the experiments, adult fish were housed in individual transparent tanks. McKay et al. designed an automated feeding system, which has several advantages over the conventional, manual handling systems: it is less invasive; it is more precise and flexible; and it can be deployed across a large number of individual tanks. It is also designed to be open-source, easily transferable, and built from only 25 widely available components.

In a proof-of-concept study, McKay et al. used their design to compare the impact of a favorable diet and a restricted diet on aging. Their results suggest that males (but not females) raised on a restricted diet live longer, and that this change is accompanied by changes in the transcriptional profiles of liver cells (Figure 1B). Sex-specific effects of diet have also been seen in mammals, which suggest that this phenomenon may be widespread among vertebrates. The sex-specific gene expression and their potential connection to lifespan differences raises many interesting questions for future research.

A critical factor in longevity studies is to study the impact of interventions on cognition. Towards this goal, McKay et al. included a red LED light in the design of their automated feeding system: this light switched on a few seconds before the fish were fed, so they can learn to associate the red light with food delivery. After a few repetitions, if the fish learned this association, they would react to the red light switching on as if they expected to be fed. This assay allowed McKay et al. to test the learning abilities and memory retention of the killifish in their home tanks, and how these were affected by age. Even though the design of the associative learning paradigm was simple, McKay et al. were able to demonstrate that killifish rapidly learned such associations in five to eight repetitions.

Overall, the study of McKay et al. opens up a number of exciting possibilities for future studies using killifish. For instance, experiments could focus on investigating how exactly dietary restriction, drug interventions, and sex-specific effects in gene expression intersect with cognitive fitness. The automated feeder should also be useful for studies looking at the effect of specific diet schedules, nutrients and circadian rhythms on longevity. Finally, as new technologies for killifish mature further (Platzer and Englert, 2016), comparative multi-species studies with other species – notably medaka and zebrafish – will become more realistic and offer the promise of even deeper insights into the biology of aging in vertebrates.

## References

[bib1] Box GEP (1976). Science and statistics. Journal of the American Statistical Association.

[bib2] Crane PA, Wilkinson G, Teare H (2022). Healthspan versus lifespan: new medicines to close the gap. Nature Aging.

[bib3] European Commission (2014). Population Ageing in Europe.

[bib4] Harel I, Benayoun BA, Machado B, Singh PP, Hu CK, Pech MF, Valenzano DR, Zhang E, Sharp SC, Artandi SE, Brunet A (2015). A platform for rapid exploration of aging and diseases in a naturally short-lived vertebrate. Cell.

[bib5] Hou Y, Dan X, Babbar M, Wei Y, Hasselbalch SG, Croteau DL, Bohr VA (2019). Ageing as a risk factor for neurodegenerative disease. Nature Reviews. Neurology.

[bib6] Kennedy BK, Berger SL, Brunet A, Campisi J, Cuervo AM, Epel ES, Franceschi C, Lithgow GJ, Morimoto RI, Pessin JE, Rando TA, Richardson A, Schadt EE, Wyss-Coray T, Sierra F (2014). Geroscience: linking aging to chronic disease. Cell.

[bib7] Mathuru AS, Libersat F, Vyas A, Teseo S (2020). Why behavioral neuroscience still needs diversity?: A curious case of a persistent need. Neuroscience and Biobehavioral Reviews.

[bib8] McKay A, Costa EK, Chen J, Hu CK, Chen X, Bedbrook CN, Khondker RC, Thielvoldt M, Priya Singh P, Wyss-Coray T, Brunet A (2022). An automated feeding system for the african killifish reveals effects of dietary restriction on lifespan and allows scalable assessment of associative learning. eLife.

[bib9] Niccoli T, Partridge L (2012). Ageing as a risk factor for disease. Current Biology.

[bib10] Platzer M, Englert C (2016). *Nothobranchius furzeri*: a model for aging research and more. Trends in Genetics.

[bib11] Reichard M, Polačik M (2019). The natural history of model organisms: *Nothobranchius furzeri*, an “instant” fish from an ephemeral habitat. eLife.

[bib12] Terzibasi Tozzini E, Cellerino A (2020). Nothobranchius annual killifishes. EvoDevo.

[bib13] Valenzano DR, Benayoun BA, Singh PP, Zhang E, Etter PD, Hu CK, Clément-Ziza M, Willemsen D, Cui R, Harel I, Machado BE, Yee MC, Sharp SC, Bustamante CD, Beyer A, Johnson EA, Brunet A (2015). The african turquoise killifish genome provides insights into evolution and genetic architecture of lifespan. Cell.

